# Acceptability and Feasibility of Longitudinal Sampling for Sexually Transmitted Enteric Infections in Gay, Bisexual, and Other Men Who Have Sex With Men (GBMSM): Prospective Cohort Pilot Study Conducted in 2022 in South East England

**DOI:** 10.2196/73762

**Published:** 2026-03-30

**Authors:** Holly Fountain, Katie Thorley, David Reid, Dana Ogaz, Daniel Richardson, Hannah Charles, Kate S Baker, Claire Jenkins, Caisey V Pulford, Gwenda Hughes, Nigel Field, Catherine H Mercer, Xavier Didelot, Noel McCarthy, Hamish Mohammed, Holly D Mitchell

**Affiliations:** 1 Blood Safety, Hepatitis, STI and HIV Division, UK Health Security Agency, London, United Kingdom; 2 University College London, NIHR Health Protection Research Unit in Blood Borne and Sexually Transmitted Infections, London, United Kingdom; 3 Institute for Global Health, University College London, Mortimer Market Centre, London, WC1E 6JB, United Kingdom, +44 2031084829; 4 University Hospitals Sussex NHS Foundation Trust, Brighton, United Kingdom; 5 Brighton and Sussex Medical School, Brighton, United Kingdom; 6 Department of Genetics, University of Cambridge, Cambridge, United Kingdom; 7 University of Liverpool, NIHR Health Protection Research Unit in Gastrointestinal Infections, Liverpool, United Kingdom; 8 Gastrointestinal Bacteria Reference Unit, UK Health Security Agency, London, United Kingdom; 9 Department of Infectious Disease Epidemiology and Dynamics, London School of Hygiene and Tropical Medicine, London, United Kingdom; 10 School of Life Sciences and Department of Statistics, University of Warwick, Coventry, United Kingdom; 11 School of Medicine, Trinity College Dublin, Dublin, Ireland

**Keywords:** sexually transmitted diseases, sexually transmitted enteric infection, gay, bisexual, or other men who have sex with men, longitudinal sampling, acceptability, feasibility

## Abstract

**Background:**

In the last 2 decades, there has been an increasing number of sexually transmissible enteric infection (STEI) outbreaks among gay, bisexual, or other men who have sex with men (GBMSM). There remain important gaps in our understanding of how STEI transmission is sustained that repeated collection of samples could help to address.

**Objective:**

This study aimed to assess the feasibility and acceptability of longitudinal samples and epidemiological data collection among GBMSM accessing sexual health services (SHS) through a prospective cohort study.

**Methods:**

GBMSM (≥16 years) accessing 2 SHS in Brighton and Sussex were recruited between May and October 2022. Participants provided an initial rectal swab and optional fecal sample and completed an online baseline questionnaire. Weekly follow-up questionnaires and rectal swabs were collected for a further 11 weeks. Sexually transmitted infection (STI) surveillance data were pseudonymously linked to provide additional clinical and demographic information. Selected participants were invited to take part in an optional one-to-one interview. We assessed the number completing study procedures, characteristics of those completing procedures and not, and representativeness of the study sample alongside facilitators and barriers from interviews. A chi-square test was used to compare groups.

**Results:**

Overall, 193 participants were recruited. Half (100/193, 51.8%) provided a baseline rectal swab, with a third (34/100, 34.0%) of them providing all 12 swabs. Alongside the baseline swab, 76.0% (76/100) provided the optional fecal sample. Just over a third (71/193, 36.8%) of participants completed a baseline questionnaire, with a fifth (15/71, 21.1%) providing all follow-up questionnaires. Interviews (n=21) found that participation was motivated by the feeling of giving something back for services received and a perceived indirect benefit to self. The study was generally accepted, with over half reflecting on the perceived ease of participation and relatively simple tasks that could be easily integrated into normal routine with an element of flexibility. Most participants were satisfied with the 12-week study length, and having a definitive end point aided the ability to assess if they would be able to participate. Barriers to completing the study procedures included not being aware of what was required, particularly for the follow-up questionnaires. Suggested improvements included concise and easier-to-read instructions, with a section to clearly list out the key procedures of the study. SMS text messaging reminders were sent, but these were seen with variable utility and interpreted in different ways (eg, personal or generic reminders or thank you messages).

**Conclusions:**

This study has provided evidence that longitudinal rectal swab sampling and data collection for research purposes are feasible and acceptable among GBMSM attending SHS. This provides an innovative way to address important knowledge gaps about STEI transmission, which will help inform public health measures for infection control. Tangible insights from this pilot can also inform the design of similar studies in other settings.

## Introduction

Enteric pathogens are classically transmitted by ingesting food or water contaminated by fecal matter, causing gastroenteritis, hepatitis, and malabsorption [1]. Over the past 50 years, it has been recognized that these pathogens can also be transmitted directly from person to person during sexual activity. Gay, bisexual, or other men who have sex with men (GBMSM) are particularly at risk of sexually transmissible enteric infections (STEIs) through sexual behaviors such as oral-anal sex (rimming), oral sex, scat play, or indirectly through oral sex after anal sex [1,2]. Transmission is likely also facilitated amongst groups of GBMSM in dense sexual networks through chemsex, group sex, and the use of sexual networking applications [3,4].

The number of reported STEI outbreaks among GBMSM globally has increased [1,5-7], with circulating strains often associated with antimicrobial resistance (AMR) [8-10], causing a growing public health concern. Antimicrobial-resistant STEIs, including *Campylobacter* spp. and Shiga toxin–producing *Escherichia coli*, and extensively drug-resistant *Shigella* spp., have all been reported, compromising management with antimicrobials [11]. Previous studies have suggested that the acquisition of AMR determinants could be linked to selection pressure from high levels of antimicrobial use in GBMSM for treating bacterial sexually transmitted infections (STIs) [8,12]. However, these mechanisms are not fully understood, and other factors, such as the gut microbiota, may also play a role in the development and severity of gastrointestinal disease and the transfer of AMR genes [13].

There also still remain important gaps in our understanding about the duration of STEI carriage and infectiousness and how this might contribute to sustained transmission of STEIs in GBMSM sexual networks. An analysis of *Shigella* spp. isolates from symptomatic adult men from the general population reported a median time interval of 23.5 days (range 6‐176 days; IQR 8‐70) between isolates recovered from the same individual [14], suggesting possible persistent, recurrent, or recrudescent infection or carriage (as no intervening clinical data were available). This highlights the need for longitudinal studies with matched questionnaire data, such as symptom status, the type or duration of reported symptoms, any antimicrobials taken, and any sexual partners or specific sexual behaviors.

Other studies have shown that asymptomatic carriage may play a key role in sustaining transmission of enteric pathogens among specific sexual networks of GBMSM [12,15,16] and may be a significant barrier to effective control. A meta-analysis provided estimates of prevalence of STEIs among asymptomatic GBMSM, ranging from 0.3% to 3.8% [17]. The analysis included 6 studies, of which only 2 explored potential risk factors, one from Melbourne, Australia, where STEI detection was associated with insertive oral-anal sex and group sex [16], and one from London, United Kingdom, where STEI detection was associated with having 5-9 new sexual partners in the last 3 months (as compared to 0-1 new partners), a bacterial STI in the last year, and the use of HIV pre-exposure prophylaxis (PrEP) or living with HIV [12]. While these studies contributed to our wider understanding of STEI transmission and the importance of certain sexual practices, they were cross-sectional and did not provide information on duration of carriage and did not include detailed information on gastrointestinal symptoms, antimicrobial treatment, or travel history.

Thus, there is a need to better understand the factors that might be sustaining transmission of, and the development of AMR in, enteric pathogens among sexual networks of GBMSM to inform the development, targeting, and delivery of clinical and public health measures that control transmission. To address these knowledge gaps, a possible study design is the repeated collection of fecal samples and/or rectal swabs, in addition to clinical and behavioral data, over a period of several months. The longitudinal samples could be sequenced and compared to study within-host evolution [18] and transmission pathways [19]. As a first step toward addressing the knowledge gaps, we conducted a mixed methods observational pilot study of sexually transmitted enteric infections among GBMSM (STEIM). We aimed to assess the feasibility and acceptability of longitudinal rectal swab sample and epidemiological data collection among GBMSM attending sexual health services (SHSs). Here we describe the characteristics of participants, completion of study procedures, and facilitators and barriers to completing the study.

## Methods

### Study Design

We conducted a longitudinal, observational prospective cohort study among GBMSM that accessed 2 SHSs in Brighton and Sussex between May 23 and October 11, 2022, with samples collected from May 26, 2022, to January 24, 2023. This region of England has high numbers of diagnoses of STEIs in GBMSM [20], making it a suitable setting for the pilot. Participants were asked to consent to provide an initial rectal swab and optional fecal sample and complete a baseline questionnaire upon enrollment. Follow-up questionnaires and rectal swabs were collected at weekly intervals for a further 11 weeks. Samples were sent to the Gastrointestinal Bacteria Reference Unit at the UK Health Security Agency (UKHSA) and tested for a range of enteric pathogens, and results were linked to questionnaire responses. Where participants provided specific informed consent, study data were linked pseudonymously (without using direct identifiers such as name or date of birth) to the national GUMCAD STI Surveillance System using unique clinic and patient numbers [21]. Selected participants were invited to take part in an optional one-to-one interview to discuss their experience of the study. These were conducted between October 2022 and March 2023.

### Study Setting

The study took place within Brighton and Sussex at SHSs provided by University Hospitals Sussex NHS Foundation Trust. In England, SHSs are free and confidential, and they are available to anyone regardless of whether the person is resident in the local area.

### Study Population

All men (cis or transgender), transgender women, or gender-diverse people, aged 16 years or older, who attended the participating SHSs during the study period and reported sex with a man (cis or transgender) or nonbinary person assigned male at birth in the previous 3 months were eligible. The study excluded those not able to provide informed consent and/or complete the study procedures due to English language or literacy. Those unable to use or access a suitable mobile device or internet were also excluded. The study aimed to recruit a representative sample of GBMSM attending the SHSs for routine STI screening or care, regardless of symptoms.

### Recruitment

All eligible participants who attended for routine sexual health care during the study period were to be approached for recruitment by SHS staff. The participation information sheet (PIS) provided for recruitment was available online during recruitment, along with the study protocol, to provide more information to potential participants.

Where eligible SHS attendees declined to participate, SHS staff were asked to complete a separate screening log to document the reasons for declining without recording personal data. Informal insights from SHS staff provided further information on the feasibility and acceptability of recruitment.

SHS staff contacted participants who agreed to be contacted about participating in a one-to-one interview. Purposive sampling was used initially to ensure representation across ages and different levels of participation in study procedures. The invite was extended to all consenting participants in the latter stages of the study.

### Sample Size

A recruitment target of 200 participants was set, based on our estimates of how many GBMSM attend the SHSs and how many will agree to participate in the time frame set for the study. No formal sample size calculation was completed, as the pilot was designed to inform sample size calculations for future larger studies. We aimed to conduct up to 20 interviews, with this estimated to be a sufficient number to reach saturation of issues related to acceptability and feasibility [22,23].

### Study Procedures

At enrollment, participants were provided with 12 rectal swabs and a fecal sample collection kit, including prepaid envelopes. For completion of the first rectal swab (the baseline rectal swab), there was the option to provide a concurrent fecal sample on the same day. After baseline, a rectal swab was to be returned each week for a further 11 weeks. Participants recorded the date of collection on the sample tube before posting.

Separate web-based questionnaires were completed at enrollment (baseline) and at each weekly follow-up. The surveys were hosted on a secure remote server (Snap Surveys Ltd) and were completed using personal digital devices. The access links to the questionnaires were available through the study web page; participants were required to enter their unique study ID only. For those who agreed to it, one-to-one interviews were also conducted to understand how best to minimize barriers and better facilitate participation and retention in the study.

For participants who gave consent, a weekly email or SMS text message reminder about the study procedures was sent on the same day every week (the day that they were recruited). Participants could choose the day of the week they completed the procedures and could continue to take part in the study even if they did not provide all the requested samples. Providing an incentive to participants was explored during the study design phase, and the decision was made not to pursue this given the study was a pilot and the aim was to assess feasibility and acceptability, including barriers and motivators for taking part. The concept of incentives was explored during the interviews.

### Data Sources and Outcomes

The data sources included behavioral and clinical data from the baseline and follow-up questionnaires; the baseline questionnaire was longer (median 8.3 minutes to complete) and had a longer look-back period than the weekly follow-up questionnaire (median 2.8 minutes to complete; Table 1 and Multimedia Appendices 1 and 2). The questionnaires did not contain any standardized tools or instruments; however, they were developed by drawing upon questionnaires used in other studies [24,25], and comprehension and clarity of the questions were checked during the study design phase.

**Table 1.** Overview of data collected and look-back periods for the baseline and follow-up questionnaires for the sexually transmitted enteric infections among gay, bisexual, or other men who have sex with men (GBMSM; sexually transmitted enteric infections among GBMSM [STEIM]) study.

Data collected	Baseline questionnaire	Look-back period	Follow-up questionnaire	Look-back period
Reason for attending SHS^a^ when recruited	Yes	N/A^b^	N/A	N/A
HIV-related factors				
HIV status and date of most recent test	Yes	No time limit	No	N/A
Antiretroviral treatment (living with HIV)	Yes	No time limit	No	N/A
Viral load count (living with HIV)	Yes	No time limit	No	N/A
HIV Pre-exposure prophylaxis (PrEP; HIV negative)	Yes	No time limit	No	N/A
Sexual behavior				
Number of sexual contacts (and additional information on partners, for example new or casual)	Yes	3 months	Yes	1 week
Type of sex	Yes	3 months	Yes	1 week
Frequency of condom use	Yes	3 months	Yes	1 week
Drug use before or after sex				
Use and frequency of listed drugs before or during sex	Yes	3 months	Yes	1 week
Injection drug use	Yes	3 months	Yes	1 week
Antibiotic use				
Consumption of antibiotics for any reason	Yes	3 months	Yes	1 week
Use of antibiotics as STI^c^ prophylaxis	Yes	3 months	Yes	1 week
Gastrointestinal symptoms				
Gastrointestinal symptoms and duration	Yes	3 months	Yes	1 week
Sexual contacts with gastrointestinal symptoms	Yes	3 months	Yes	1 week
Other contacts (eg, household) with gastrointestinal symptoms	Yes	3 months	Yes	1 week
Travel				
Travel outside the United Kingdom	Yes	3 months	Yes	1 week
New sexual partners while abroad	Yes	3 months	Yes	1 week

a SHS: sexual health service.

b N/A: not applicable.

c STI: sexually transmitted infection.

One-to-one interviews were conducted using a semistructured topic guide. This was developed collaboratively and designed to allow for data on participation motivation, research experience, understanding of compliance with and the acceptability of study requirements and procedures, barriers and facilitators to study participation and retention, and suggestions for improvement for subsequent research to be collected. Interviews were conducted by one member of the STEIM study team (D Reid) and were conducted as remote audio or video interviews using Microsoft Teams. All interviews were also transcribed through Microsoft Teams, with transcriptions reviewed by the interviewer. As this was a pilot study, interviews were not piloted beforehand.

Additional clinical and demographics information was sourced from GUMCAD. GUMCAD is the mandatory surveillance system for STIs in England and collects data on STI tests and diagnoses from all publicly commissioned SHSs [21]. For consenting participants, pseudonymous linkage to GUMCAD data was conducted to provide additional clinical and demographic information, including recent STI diagnoses (*Chlamydia trachomatis, Neisseria gonorrhoeae,* or *Treponema pallidum* [primary, secondary, or early latent] at enrollment and in the past 12 months); HIV status; HIV PrEP use; sexual orientation; ethnicity and country of birth; and patient residence to allow for determination of index of multiple deprivation (IMD).

Outcomes of the study included the number and proportion of participants providing samples and questionnaires at various time points throughout the study, for example, how many completed baseline procedures and how many completed all weeks; description of the characteristics of the study population; comparison of the characteristics of participants who did and did not return samples and questionnaires; whether the study population was representative of all GBMSM attending the SHSs during the study period; and facilitators and barriers to the study as drawn out from the one-to-one interviews. Qualitative interview results were used to contextualize the quantitative results and to provide a full picture of the acceptability and feasibility of the pilot study.

### Data Management and Analysis

All quantitative data were managed and analyzed using Microsoft Excel and STATA (v17; StataCorp LLC). Qualitative data were analyzed using NVivo (20; Lumivero).

GUMCAD records were linked to the enrollment log using a deterministic linkage approach based on participants’ patient number, SHS attended, and SHS attendance date. Data matches were accepted where the GUMCAD SHS attendance date was within 30 days of the participant enrollment date, but information on concurrent STI diagnosis at baseline was only included where the dates matched exactly. After records were linked, the GUMCAD patient number was dropped. The GUMCAD data were subsequently linked to the laboratory testing results and questionnaire responses using the study ID. Where responses from the questionnaire differed from GUMCAD data on the same topic, the questionnaire response was used for analysis (being the most up-to-date and less subjective to clinician or coder bias) and replaced the input from GUMCAD. When the date of collection of rectal swabs was not provided (254/949, 26.8%), the date of receipt at UKHSA was used.

Summary metrics, such as the median number of samples returned, were used to assess the feasibility of longitudinal rectal swab collection and completion of self-administered questionnaires. Participant characteristics were analyzed and summarized using data from GUMCAD and questionnaires. To minimize the risk of deductive disclosure, in some cases, small numbers (1-4 inclusive) were masked and referred to as “<5” or groups containing small numbers have been aggregated. The characteristics of participants enrolled in the study who did and did not return at least one sample or completed questionnaire were compared using the chi-square test.

The representativeness of the study sample was ascertained by comparing the demographic characteristics, obtained from the GUMCAD data, to participating SHS attendees during the study period, excluding the study participants, who reported as GBMSM; the chi-square test was used for statistical comparisons.

An analytic “framework” approach [26] was used to analyze the interview transcripts. Initial deductive and inductive thematic codes were derived using the interview topic guides and 3 transcripts by 2 members of the STEIM study team (D Reid and DO). Wider themes were discussed to agree on all themes and coding structure before the agreed codes were then applied to all transcripts (by D Reid) and data were charted, summarized, and interpreted. As this was a pilot study, we did not formally assess saturation, and the number of interviews was set in advance.

### Ethical Considerations

This study was reviewed and approved by the London and South East NHS Research Ethics Committee (Reference 21/LO/0891) and the University Hospitals Sussex NHS Foundation Trust Research and Development department. Written informed consent was obtained from all participants prior to participation (Multimedia Appendix 3), and participants could withdraw from the study at any time. Those willing to take part in an optional one-to-one interview completed a separate interview consent form (Figure S1 in Multimedia Appendix 4). In addition, consent for (1) linkage to GUMCAD data, (2) residual study samples to be stored for future ethically approved research studies, (3) text or email reminders to be sent about completing the follow-up procedures each week, and (4) participating in the one-to-one interview was recorded. All four of these tasks were optional to agree to. All data were pseudonymized using a unique study identifier before sending to researchers, so they did not have access to personal identifying information. No images are provided in the manuscript or supplementary material that would allow identification of individual participants. No type of incentive was given to participants.

During the study design phase, cognitive interviews with GBMSM were used to explore general interest and acceptability of the study proposal and methodology as part of the patient and public engagement and involvement strategy. The interviews also explored acceptability, comprehension, and clarity of the participant information documents and questionnaires. Suggestions for improvement to methods and study materials were incorporated.

## Results

### Quantitative Results

#### Recruitment and Study Completion Rates

Overall, 193 people were recruited into the STEIM study between May 23, 2022, and October 11, 2022 (96.5% of the planned 200 to be recruited). Screening logs were completed for 23 eligible SHS attendees who did not agree to take part; not having the time or capacity to participate was cited as a reason by half (12/23), often due to travel abroad or relocating (7/12). Others perceived they had too few recent sexual partners or had not engaged in specific sexual behaviors to be suitable for the study (7/23), lacked access or proficiency using the internet (n<5), or did not want to provide the samples required (n<5). Half (12/23, 50%) of the screening log entries were from the Sussex SHS. Informal discussions with SHS staff indicated that the screening log was not routinely completed, meaning a lack of accurate records of those declining to participate. They also highlighted that the most frequently heard refusal was a lack of interest in participating. Other reasons included being put off by rectal swabbing and the lack of returned diagnostic results.

Most participants were recruited at the SHS in Brighton (168/193, 87.0%), the largest SHS in the study area. Baseline questionnaires were completed by 71 participants (71/193, 36.8%), among which most were recruited while attending for a vaccination (28/71, 39.4%), a general sexual health check (22/71, 31.0%), and/or to receive HIV PrEP (20/71, 28.2%). Informal discussions with SHS staff suggested that recruitment was often conducted during mpox vaccination clinics (the opening of recruitment coincided with the start of the mpox outbreak in the United Kingdom in the summer of 2022).

**Figure 1.**
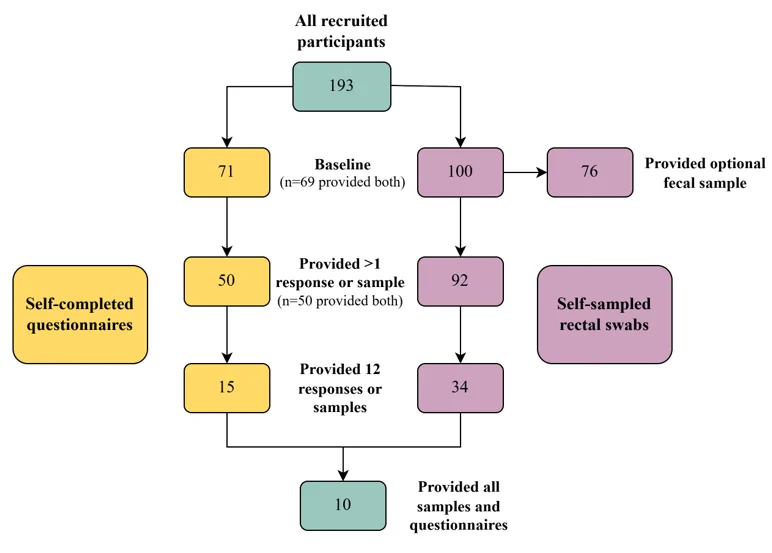
Flowchart of sexually transmitted enteric infections among gay, bisexual, or other men who have sex with men (GBMSM; sexually transmitted enteric infections among GBMSM [STEIM]) study participant recruitment and participation by study component (self-completed questionnaires and self-sampled rectal swabs and fecal sample).

Of those recruited, 51.8% (100/193) provided a baseline rectal swab (Figure 1), among which 34.0% (34/100) provided all 12 swabs (Figure S2 in Multimedia Appendix 4 shows more details), with a median of 11 rectal swabs submitted (IQR 5-12). Those providing all 12 swabs did so over 11-23 weeks, with 9 participants providing swabs for 14 or more weeks. Among these participants, 76.0% (76/100) also provided an optional fecal sample. Of those who completed a baseline questionnaire, 21.1% (15/71) completed all follow-up questionnaires (Figure 1) over 11-15 weeks. There were 11 participants who submitted follow-up questionnaires but no baseline; these participants were not included in completed questionnaire counts, and most submitted 3 or fewer questionnaires (7/11, 63.6%), with only one submitting all follow-up questionnaires.

#### Feasibility of Data Linkage

Most participants (188/193, 97.4%) agreed to GUMCAD data linkage, and linkage was successful for 97.9% (184/188). Of the 102 participants with a baseline sample and/or questionnaire, 98.0% (100/102) agreed to linkage, and 98% (98/100) were successfully linked to a GUMCAD record. Demographic data were obtained for all of those successfully linked; concurrent STI diagnosis at baseline was available for 97.3% (179/184) of participants.

#### Characteristics of Study Participants

Most study participants were UK-born (141/184, 76.6%) and of white ethnicity (171/184, 92.9%; Table 2), with a median age of 43 years (IQR 33-52). At baseline, most participants were not known to be living with HIV (134/184, 72.8%), of which 47.8% (64/134) were taking HIV PrEP. While only 5.0% (9/179) of participants were diagnosed with a bacterial STI on the day of enrollment, 38.0% (70/184) had a bacterial STI diagnosed in the previous year.

**Table 2.** Characteristics of sexually transmitted enteric infections among gay, bisexual, or other men who have sex with men (GBMSM; sexually transmitted enteric infections among GBMSM [STEIM]) study participants compared to all GBMSM sexual health service (SHS) attendees (GUMCAD national sexually transmitted infection [STI] data) between May 2022 and October 2022.

Characteristics	Study participants^a^^,^^b^ (n=184)		SHS^c^ attendees, excluding study participants (n=4238)		*P* value
Age (years), n (%)					.06
16-24	6 (3.3)		339 (8.0)		
25-34	47 (25.5)		913 (21.5)		
35-44	44 (23.9)		987 (23.3)		
45-64	79 (42.9)		1688 (39.8)		
≥65	8 (4.3)		311 (7.3)		
Unknown/missing	0 (0)		0 (0)		
Sexual orientation, n (%)					N/A^d^
Heterosexual	1 (0.5)		N/A		
Gay	164 (89.1)		N/A		
Bisexual	9 (4.9)		N/A		
Other, not listed	10 (5.4)		N/A		
Unknown	0 (0.0)		N/A		
Ethnicity^e^, n (%)					.12
White	171 (92.9)		3736 (88.2)		
All other ethnic groups	9 (4.9)		387 (9.1)		
Unknown/missing	4 (2.2)		115 (2.7)		
Region of birth^f^, n (%)					.67
United Kingdom	141 (76.6)		3222 (76.0)		
All other regions of birth	43 (23.4)		998 (23.5)		
Unknown	0 (0.0)		18 (0.4)		
Index of multiple deprivation (IMD) quintile, n (%)					.94
Most deprived	34 (18.5)		807 (19.0)		
2	43 (23.4)		1041 (24.6)		
3	52 (28.3)		1126 (26.6)		
4	34 (18.5)		725 (17.1)		
Least deprived	16 (8.7)		444 (10.5)		
Unknown/missing	5 (2.7)		95 (2.2)		
HIV status with PrEP^g^ status, n (%)					<.001
Not known to be living with HIV, taking PrEP	64 (34.8)		608 (14.3)		
Not known to be living with HIV, not taking PrEP	70 (38.0)		1854 (43.7)		
Living with HIV	50 (27.2)		1776 (41.9)		
STI in the previous year^h^, n (%)					<.001
Yes	70 (38.0)		862 (20.3)		
No/unknown	114 (62.0)		3376 (79.7)		
Concurrent STI^i^ (ie, STI diagnosed on day of enrollment)^h^, n (%)	n=179				N/A
Yes	9 (5.0)		N/A		
No/unknown	170 (95.0)		N/A		

a Data obtained from routine returns to the GUMCAD STI surveillance system and baseline questionnaire entries, where eligible for the study participants only.

b Study participants were eligible if reporting sex with a man (cis/transgender) or nonbinary person assigned male at birth in the past 3 months; as this same level of detail on sexual partners is not available in GUMCAD, the comparison is made to all male individuals with sexual orientation reported as gay or bisexual in GUMCAD data rather than explicitly those reporting sex with a man in the past 3 months.

c SHS: sexual health service.

d N/A: not applicable.

e All other ethnic groups include groupings for those of Asian ethnicity, Black ethnicity, mixed ethnicity, and any other ethnicity. These data were aggregated into one group due to small data points.

f All other regions of birth include groupings for those born in the Caribbean, Central and South America, North America, Europe, South Asia, sub-Saharan Africa, and any other region. These data were aggregated into one group due to small data points.

g PrEP: pre-exposure prophylaxis.

h Concurrent or previous STI was considered as *Chlamydia trachomatis*, *Neisseria gonorrhoeae,* or *Treponema pallidum* (primary, secondary, or early latent) diagnosis.

i STI: sexually transmitted infection.

From the baseline questionnaires, the most common number of sexual partners in the previous 3 months was between 2 and 4 (29/71, 40.8%; Table 3). During that time, very few participants had used STI prophylaxis (taken antimicrobials immediately before or after sex to prevent STIs other than HIV; 2/71, 2.8%; missing data n=1, 1.4%), and a majority of participants (69.0%, 49/71) reported gastrointestinal symptoms, with diarrhea being most common (42/49, 85.7%) followed by mucus and/or blood in feces (23/49, 46.9%). Just under half (31/71, 43.7%) had traveled abroad, and about a third (10/31, 32.3%) of those reported sex abroad.

**Table 3.** Sexually transmitted enteric infections among gay, bisexual, or other men who have sex with men (GBMSM; sexually transmitted enteric infections among GBMSM [STEIM]) study participant behavioral characteristics, from baseline questionnaires between May 2022 and January 2023.

Characteristics	Number of participants (n=71), n (%)	
Number of sexual partners (past 3 months)		
1	7 (9.9)	
2‐4	29 (40.8)	
5‐10	18 (25.4)	
11‐19	4 (5.6)	
20‐99	11 (15.5)	
Don’t know	2 (2.8)	
STI^a^ prophylaxis		
No	68 (95.8)	
Yes	2 (2.8)	
Don’t know	1 (1.4)	
Travel abroad		
No	40 (56.3)	
Yes	31 (43.7)	
Don’t know	0 (0.0)	
Sex abroad		
No	19 (61.3)	
Yes	10 (32.3)	
Not answered	2 (6.5)	
Not applicable	40	
GI^b^ symptoms (past 3 months)		
No	22 (31.0)	
Yes	49 (69.0)	
Don’t know	0 (0.0)	

a STI: sexually transmitted infection.

b GI: gastrointestinal

Nearly half (89/193, 46.1%) of participants did not complete questionnaires or provide samples after initial recruitment. These participants had similar characteristics to those who did provide samples and/or questionnaires (Table S1 in Multimedia Appendix 4). However, participants who provided completed samples or questionnaires were more likely to be from the least deprived IMD quintiles (*P*=.004). Participants returning samples or questionnaires, and who were not known to be living with HIV, were more likely to be taking HIV PrEP (46.5% vs 21.2%; *P*=.001) and less likely to be diagnosed with a concurrent bacterial STI at enrollment (2.1% vs 8.3%; *P*=.06).

#### Representativeness of Study Population

Recruited participants were representative of GBMSM attending the participating SHSs within the study period for most characteristics. Most GBMSM attending the participating SHSs were also UK-born (76.0%) and of White ethnicity (88.2%; Table 2), with a median age of 43 (IQR 33-54) years. However, more study participants were taking HIV PrEP (34.8% vs 14.3%; *P*<.001) and more had experienced a bacterial STI in the previous year (38.0% vs 20.3%; *P*<.001) compared to all GBMSM attending the SHSs.

### Qualitative Results

#### Characteristics of Interview Participants

Most participants (153/193, 79.3%) agreed to be contacted for an interview, with 21 participants completing interviews. Interviewees were slightly older on average than all participants, ranging from 28 to 76 years old with a median of 49 (IQR 43-55) years, compared to a median of 43 (IQR 33-52) years for all participants. Most interviewees reported some pre-STEIM research participation (17/21), and they had better completion of study procedures than the overall study participants, with almost all interviewees submitting a fecal sample (20/21) and all submitting one or more rectal swabs.

#### Motivations for Study Participation

Qualitative findings from interviews showed that the context in which participants were recruited may have motivated many to consider joining the study, with most interviewees mentioning concurrent receipt of mpox vaccination or other valued services:

*I’m really grateful for having the monkeypox vaccine so quickly and because of that, I’m going to take part in this slightly uncomfortable study as my way of giving something back.*

Taking part in STEIM was perceived as a welcome opportunity for many to give back to SHSs.

Almost all participants referred to indirect benefits to self through helping others or achieving a collective benefit for peers. More than half (12/21) of the interviewees suggested they were interested in the STEIM study or studies related to gut health or the sexual health of gay men more generally. The relative novelty of a sexual health study focused on gut infections was appealing and interesting to many. Two participants did mistakenly expect that tests on their samples that identified an infection would be communicated either to medical staff or themselves and therefore incorrectly inferred a potential direct benefit in early diagnosis and potential treatment.

All interviewees had some initial discussion with one or more SHS staff members before agreeing to participate. Staff members who were known, trusted, and enthusiastic about the study encouraged participation. Reassurances that tasks were not onerous and perceptions of staff as knowledgeable and able to provide ongoing support or advice also promoted a decision to participate.

Many participants noted that an incentive to take part would not personally be a motivator to take part in the study, but the acceptability of an incentive offer was reported by a majority. Financial incentives were mentioned, and it was suggested that these should be easy to use, useful, small, and targeted at those in need. Some participants suggested they may also be incentivized by receiving study results instead of a monetary reward; from one participant,

*I’m never going to say no to money, but it’s ok if someone wants to give you money for the thing. But I’d be happy if it was just a general, either like a feedback, personal health monitoring thing or seeing the sort of results of the study. So, seeing what it’s accomplishing, hearing sort of updates on what the research is finding*.

#### Facilitators and Barriers to Participation and Completion of Procedures

Over half of interview participants expressed no reservations about participating in the study, reflecting on the perceived ease of participation and relatively simple and not onerous tasks. From one participant,

*Once you get into it, it was very straightforward and easy. You know, I think for me; do the sample, fill in the survey, 5 minutes, not an issue, you know and, tick the box and job done. Really.*

Study time requirements and potential disruption to normal life were important considerations for more than half, though generally assessed as acceptable. A few participants related that a definite study end point aided them in assessing the time required when considering other commitments such as work or holidays. Most were highly satisfied with the 12-week pilot length, a majority (16/21) of whom felt it was a preferable maximum. Three months is a period most felt they could commit to participate and in which interest and remembering tasks could be maintained. From one participant, “*Probably about this [length] would have been the longest. I wouldn’t have done it if it was much more*.” However, half the participants (11/21) would consider a longer study duration, and a third reported that they would consider 6 months to 1 year, and a small minority may consider an indefinite period.

The ability to integrate and routinize the study into participants’ lives facilitated participation. The flexibility to choose a preferred day of the week to complete the study procedures, with allowances and leeway of a day or 2, was appreciated and reported as useful. Many participants recounted the ease of taking rectal swabs as part of their morning routine or in posting samples soon after on the way to work, during a walk, or when passing a postbox. The weekly repetition of tasks helped to aid memory, and over half the participants discussed the utility of setting their own reminders, alarms, or in-diary tasks positively.

Many discussed the variable or limited utility of text reminders sent by the clinic. These texts sometimes came on a different day from that set by the participant and were perceived in different ways as reminders, either targeted personally or automatic and generic or as thanks or acknowledgment of completion and receipt of samples. These interpretations were influenced by whether they arrived before or after the day that samples were taken, posted, or received and by previous interpretations or expectations. The utility of these texts was enhanced if they were seen to improve a sense of study connection and engagement, which participants suggested might be increased by a personal rather than generic focus, being tailored to tasks and including messages of appreciation for participation as well as feedback on study findings.

Reception to the PIS and other written instructions was mixed, with participants often suggesting improvements to these. In reading the PIS, some participants reported that they missed aspects of the study methodology or were left uncertain and made suggestions for step-by-step instructions or bullet points of the key tasks.

*I think the way that it’s all presented, a patient information sheet, it’s quite sort of quite a lot of dense information and lots of separate paragraphs. […] Just too much text and I think I could have done with a little reminder right at the top, maybe at the top of that just bullet points like every week, one bullet saying take your swab and send it off and then another bullet point saying complete the questionnaire.*

#### Facilitators and Barriers to Rectal Swab and Stool Sample Collection

The ability to provide the specimens at home, allowing for privacy and saving time and potential SHS visits, was appreciated and desired. One interviewee stated,

*… I think the fact that I could do it myself and post them in was one of the things that convinced me it would be fine to do it*.

Interviews suggested that there were differences in the interpretation of the end point of the study; most interviewees reported believing that the end point was when all swab samples were submitted, but others identified 12 weeks as an end point even if some samples were missed, which was the intended outcome.

Instructions for rectal swab collection were described as useful for understanding procedures, and those for stool sample collection detailed best practices, though many men described using methods of collection that were slightly adapted to suit their own situation and resources, for example, using a toilet paper nest or a Tupperware container. Most interview participants were happy to provide rectal swabs, relating a perception of rectal swab collection that was relatively simple.

There was less general acceptance for the fecal samples. Interview participants perceived stool sample collection as more difficult than rectal swabbing. They discussed difficulties, such as disgust in handling feces, less familiarity with collection, requirements to follow body processes and timings, and more complexity in planning and execution. This included needing a private toilet and more complex requirements for preparing and taking the sample, getting the sample into the correct container in the required amount without contamination, and disposing of or cleaning equipment or containers. Overall, a quarter of participants (5/21) felt it was a difficult thing to do; the rest were relatively comfortable while also acknowledging less ease and greater effort were required than in taking rectal swabs [1].

#### Facilitators and Barriers to Questionnaire Completion

Interviews confirmed that the aspect of the study that proved the most challenging or difficult to complete as instructed was the online weekly questionnaires. No participant reported returning a questionnaire on the same day every week for a consecutive 12-week period, and almost a third (6/21) reported being unaware of a requirement to complete a weekly questionnaire at all. From one participant,

*Oh, I didn’t get that because I think when I read and I got information, I thought there only was one questionnaire at the start of the study and then you only send samples*.

Some of the men interviewed were not aware of online questionnaire requirements, though they reported reading participant information detailing them. Suggestions were made for improved instructions, including greater clarity on requirements for weekly questionnaire completion, less densely presented but more concise information, bullet points with study requirements, and use of less formal and more readable language.

Among participants who completed the questionnaires, this was facilitated by online links or QR codes in study information, text reminders, or self-bookmarking the site of the weekly questionnaire. From one interviewee,

*[…] But then I got a text that day thanking me for taking part in the study which had the link embedded which is really useful because I couldn’t remember what the hell the name of it was*.

The content and length of the questionnaires were largely acceptable to those who were aware of and completed one or more questionnaires.

*The length as in. Uh, yeah [...] I found it pretty straightforward and easy, in fact, I wish I’d known about it earlier. I would have probably done it more regularly if I’d known how easy it was*.

Overall, participants expressed comfort with the content of questions, and their assessments suggested a reasonable understanding or acceptance that questions were included because they would be useful and appropriate to the study.

A couple of interviewees related some irritating repetition in individual surveys or in having to answer questions when little had changed in their behavior from preceding weeks and may prefer a more concise way of indicating as such, for example, a “No change” option that would result in skipping most of the questions. Others suggested they would appreciate improvements to layout and legibility, particularly on mobile screens.

*[…] I tried to do [the questionnaire] on my phone again, but it was just impossible. It was just far too small*.

## Discussion

The STEIM study design was feasible to implement and largely acceptable in an SHS-attending GBMSM population in Brighton and Sussex. The study team was successful in recruiting a study population of the target size that was broadly representative of GBMSM attending the participating SHSs. Just under half of participants provided more than one rectal swab, and many provided weekly or nearly weekly swabs. Questionnaire completion was less complete than swab return. Insights from qualitative interviews identified barriers and facilitators that could be addressed in a future implementation of the study design, and many made suggestions for improving participant-facing study materials. For example, participants reported not being aware of the need for regular questionnaire completion and being surprised about how easy they were to complete, which could be addressed by improved study information and communication.

Our findings align with other research involving the recruitment of GBMSM to provide self-completed questionnaires and a biological sample; one study recruiting GBMSM at a central London SHS to complete a questionnaire and collect biological samples for human papillomavirus testing had a high acceptance rate of 81.4% [27], while the Gay Men’s Sexual Health Survey in Scotland recorded a response rate of 65.2% for the survey aspect, with 80.4% of those surveyed providing an optional oral fluid specimen [28]. Our findings are particularly encouraging given the longitudinal sampling component of STEIM.

Qualitative interviews highlighted that the innovation of the study, especially given the focus on gut health, was appealing to many and may have helped with recruitment. Altruism is a common facilitator for participation in a study [29], and the feeling of giving something back was also a driver for participation in this study, with some recruitment happening in vaccination clinics responding to the 2022 mpox outbreak in England. This was a special circumstance, but linking recruitment to valued services may be beneficial for future studies.

Perceived personal benefits are also a common facilitator [29]. However, the study design did not provide individual sample test results, which could present a possible barrier to participation. Two participants reported being motivated to participate because they expected to receive diagnostic test results. It will be important to better emphasize that this is not the case for future recruitment or to develop mechanisms to explore feedback in future studies, especially as it was suggested that receiving results, both individual sample and overall study results, may incentivize participants to complete the study.

A large proportion of participants did not complete any study procedures after enrollment. Other studies have documented similar problems. For example, in Positive Voices, a national survey of people living with HIV, 90% of individuals accepted the initial survey invitation, but only 23% completed it [24]. Unfortunately, limited data were available to understand the drop-off in this study, with no qualitative interviews conducted with these participants. Demographic analysis showed that those who completed study procedures were more likely to have taken HIV PrEP or to live in the least deprived IMD quintile, highlighting more routine use of SHSs; a desire for reciprocity of valued services or high socioeconomic status might facilitate taking part. Future studies must address this challenge, potentially through better public engagement work or through improving the study procedures based on feedback from this pilot. While the lack of financial offers was not highlighted as a barrier to participation in the interviews, with no interviews conducted with those who did not complete procedures, it is possible that financial incentives could help give higher overall participation and retention for future studies, especially for those living in more deprived areas, who were less likely to complete study procedures. The potential drop-off should also be accounted for in future sample size calculations.

Participant engagement with questionnaires was challenging, with 36.8% of participants completing a baseline and only 15 participants completing all follow-up questionnaires, less than half the number who provided rectal swabs for the whole study. Qualitative interviews revealed low awareness of questionnaires, especially the requirement for weekly completion, and this will be a focus for future iterations. Concise, bulleted instructions of the key steps might help improve participant understanding and completion rates. Optimizing mobile access and completion of the questionnaire could also improve engagement, for example, by using the mobile surveys feature of Snap Surveys Ltd.

SMS text messaging sent to participants as reminders to complete study procedures had variable utility, with mixed understanding as to whether they were reminders or were a thank you instead. A meta-analysis showed varied usefulness of reminders in promoting participant engagement with online surveys [30]. The utility of SMS text messaging increased if they were seen to improve a sense of study connection and engagement, which participants suggested might be done by a more personal focus and tailoring to specific tasks. In other studies, personalized reminder messages have been discussed as a useful tool to generate extra responses and encourage reluctant participants [31].

There was better engagement with the return of rectal swabs, with over half of those returning one swab returning 10 or more swabs. This might be because rectal swab sampling was easier to incorporate into daily routines, as highlighted by qualitative interviews. Qualitative interviews also revealed that most participants were satisfied with the 12-week study length and that the definitive end point supported decision-making on the ability to participate, further suggesting that this type of longitudinal sampling is feasible. Patient familiarity with the rectal swab procedure, a routine form of testing in SHS [32], may also have contributed to acceptability in this cohort.

Even so, few of the interviewees at the time of the interview had fully completed the study procedures, and none completed them on the same day every week. This indicates that, although feasible and acceptable, competing life tasks and the unpredictability of weekly routines still limit participation in a longitudinal study. It will be important to ensure that participants are aware there is flexibility and that they can complete weekly procedures on different days or miss one week (or a couple) and still provide important participation.

There are some limitations to the STEIM study. Regarding recruitment, estimating recruitment success relied on the completion of study screening logs, which were not routinely done, leading to possibly overestimated recruitment success. Participants were required to have sufficient English language and literacy skills and access to a mobile device and the internet to be able to complete the study procedures, and most participants were recruited from Brighton SHS, making this essentially a single-center study. Together, this limits the generalizability of the study, the demographics of the recruited individuals, and the participant experience. Based on experience from other studies [25], we were confident that online data collection methods were acceptable to most SHS attendees in this population, and within the time and financial constraints of our pilot study, it was not possible to translate materials into different languages or supply alternatives to digital devices. However, these options should be explored for future studies to increase the generalizability of the study and the representativeness of those who complete study procedures. Additionally, the STEIM study had specific eligibility criteria, such as having had a recent sexual partner, which, at that time, could not be compared to national STI surveillance data. Therefore, representativeness was compared to the overall population and may explain the higher frequency of recent STIs and taking HIV PrEP in the study cohort. Interview participants had all returned at least one questionnaire and rectal swab, which limited our qualitative insights on barriers to completing study procedures.

Overall, based on the findings from this pilot study, we recommend that future studies (1) provide a user-friendly outline of the study requirements at the beginning of the participant instruction sheet that clearly lays out the expectation of the participant over the study period through concise bulleted instructions, with an emphasis on the weekly questionnaire alongside the swab; (2) modify SMS text messaging reminders so that they improve a sense of connection to the study, for example, are tailored to specific tasks, and are clearly phrased to serve as reminders of study procedures and what those procedures are; (3) optimize the questionnaires for use on mobile phones; (4) consider participation incentives, which could include financial incentives and/or reports on study results; and (5) keep and emphasize the element of flexibility to the study procedures so that they can be integrated into normal routine.

In conclusion, this phase of the STEIM study has provided evidence of the feasibility and acceptability of the study methods, as well as tangible insights to inform the design of future similar studies involving longitudinal rectal swabs and self-reported data collection for STEI-related studies as well as for studies in other sexual health patient populations and outside of this setting. This pilot has provided innovative results in the field of STEIs; previous research studies for exploring risk factors for and transmission of STEIs (in the United Kingdom) have been cross-sectional in nature [12,15] and, to our knowledge, this is the first study that is longitudinal. This type of study is needed to address important gaps in our understanding of STEI transmission, which can then be used to inform appropriately targeted clinical and public health interventions for infection control.

## Supplementary material

10.2196/73762 Multimedia Appendix 1 Study baseline questionnaire.

10.2196/73762 Multimedia Appendix 2 Study follow-up questionnaire.

10.2196/73762 Multimedia Appendix 3 Study participant consent form.

10.2196/73762 Multimedia Appendix 4 Additional figures and table.

## Abbreviations

AMR: antimicrobial resistance
GBMSM: gay, bisexual, and other men who have sex with men
IMD: index of multiple deprivation
PIS: participant information sheet
PrEP: pre-exposure prophylaxis
SHS: sexual health service
STEI: sexually transmitted enteric infection
STEIM: sexually transmitted enteric infections among GBMSM
STI: sexually transmitted infection
UKHSA: UK Health Security Agency
