# Supplementary material for: Acceptability and Feasibility of Longitudinal Sampling for Sexually Transmitted Enteric Infections in Gay, Bisexual, and Other Men Who Have Sex With Men (GBMSM): Prospective Cohort Pilot Study Conducted in 2022 in South East England
Source: JMIR Public Health Surveill. 2026 Mar 30;12:e73762. doi: 10.2196/73762 (PMC13035075; doi:10.2196/73762)
Supplement: Multimedia Appendix 1 [file publichealth-v12-e73762-s001.pdf]

<u>STEIM PILOT STUDY BASELINE QUESTIONNAIRE</u> (Multimedia appendix)

This is a Multimedia Appendix to a full manuscript published in the J Med Internet Res. For full copyright and citation information see http://dx.doi.org/10.2196/jmir.73762

Thank you for agreeing to take part in this study. The study will help us to understand the extent to which gut bugs (e.g. *Shigella*) are spreading, to identify who may be at higher risk of being infected and how we can best treat the infections.

Please complete this questionnaire on the same day as you collect your first rectal swab and your poo sample. Providing a poo sample will tell us a lot about the types of bugs in the gut. You can opt-out of providing a poo sample if you prefer.

The questionnaire will ask you some personal questions. We are asking these questions because they will help us to better understand how the bugs are spreading between people. You can skip any questions you do not want to answer. All answers are strictly confidential.

Please enter your Study ID:

Please enter today's date:

Part 1: Clinic attendance

Q1.1 Why are you attending the clinic today? [Tick all that apply]

- Want a general sexual health check-up
- No symptoms, but was worried might have an STI
- Have symptoms
- A sexual partner had symptoms
- A sexual partner was diagnosed with an STI
- Treatment after a previous positive STI test
- Check-up after a previous positive STI test
- As follow-up to an online test
- To get Pre-Exposure Prophylaxis (PrEP) to prevent HIV (the use of antiretroviral drugs **before** sex to reduce the risk of HIV infection)
- To get Post Exposure Prophylaxis (PEP) to prevent HIV (the emergency use of antiretroviral drugs **after** sex to reduce the risk of HIV infection)
- HIV care and treatment
- Advice or counselling
- I needed condoms
- I needed a vaccination
- I couldn't get an online testing kit
- I was told to attend by my GP/family doctor or another healthcare professional
- Other [please specify]

Part 2: HIV

Q2.1 What was the result of your last HIV test?

- Never tested **(Route to Q2.1.4)**
- HIV positive **(Route to Q2.1.1)**
- HIV negative **(Route to Q2.1.3)**
- Don't know **(Route to Q2.1.4)**

Q2.1.1 Are you currently on antiretroviral medication (ART or HAART) for your HIV infection?

- Yes
- No

Q2.1.2 What was the result of your last viral load test (copies per ml)?

- More than 200
- 40-200
- Less than 40 (undetectable)
- Not sure/Don't remember

(Route to Q3)

Q 2.1.3. When did you last have an HIV test before today?

- Less than 1 month ago
- 1 to 3 months ago
- 3 to 6 months ago
- 6 to 12 months ago
- More than 1 year ago

Q2.1.4 Are you currently taking PrEP (the use of antiretroviral drugs before sex to reduce the risk of HIV infection)?

- Yes
- Not currently, but I have used PrEP in the past 3 months
- Not currently, but I have used PrEP in the past (over 3 months ago)
- I have never used PrEP, but I am aware of what PrEP is
- I do not know what PrEP is

Part 3: Sexual behaviour

This section asks about your sexual partners and recent sexual history. Please answer the questions as best as you can

Q3.1 In the past 3 months, how many cis men (men assigned male at birth), trans men, trans women or gender-diverse people have you had any physical sexual contact with?

(By sexual contact, we mean any activity intended to achieve orgasm (or close to orgasm) for one or both partners).

- 1
- 2-4
- 5-10
- 11-19
- 20-49
- 50-99
- 100+
- Don't know

Q3.1.1 How many of those partners in Q3.1 were new partners that you had sex with for the first time in the past 3 months?

- 0
- 1
- 2-4 (shown if Q3.1 is 2-4 or greater)
- 5-10 (shown if Q3.1 is 5-10 or greater)
- 11-19 (shown if Q3.1 is 11-19 or greater)
- 20-49 (shown if Q3.1 is 20-49 or greater)
- 50-99 (shown if Q3.1 is 50-99 or greater)
- 100+ (shown if Q3.1 is 100+)
- Don't know

Q3.1.2 How many of those new partners in Q3.1.1 have you only had sex with once and probably will not have sex with again? (e.g. one-night stands, cruising partner)

- 0
- 1
- 2-4 (shown if Q3.1 is 2-4 or greater)
- 5-10 (shown if Q3.1 is 5-10 or greater)
- 11-19 (shown if Q3.1 is 11-19 or greater)
- 20-49 (shown if Q3.1 is 20-49 or greater)
- 50-99 (shown if Q3.1 is 50-99 or greater)
- 100+ (shown if Q3.1 is 100+)
- Don't know

We'd now like to ask a bit more detail about the type of sex you have had. Please look at the questions below and answer as best as you can.

	**Q3.2 In the past 3 months, what types of sex have you had?** (tick all that apply)	**Q3.2.1 In the past 3 months, how often have you used condoms at all times when having this type of sex?** (Shown if Q3.2 is ticked)	**Q3.2.2 In the past 3 months, how many men (cis/transgender), trans women or gender-diverse people have you had this type of sex with?** (Shown if Q3.2 is ticked)	**Q3.2.3 When did you last engage in this type of sex?** (Shown if Q3.2 is ticked)
Anal sex (insertive/active/top)<br>*(Your penis enters another person's anus)*		Always<br>Sometimes<br>Never	1, 2-4, 5-10, 11-19, 20-49, 50-99, 100+. Selection shown based on answer to Q3.1.	Less than 1 week ago<br>1-2 weeks ago<br>2-4 weeks ago<br>More than 4 weeks ago
Anal sex (receptive/passive/bottom)<br>*(Another person's penis enters your anus)*				
Receptive anal sex followed by active oral sex<br>*(Another person's penis enters your anus, then your mouth contacts the other person's penis)*				
Insertive anal sex followed by receptive oral sex<br>*(Your penis enters another person's anus, then their mouth contacts your penis)*				
Fisting (active)<br>*(Your fist enters another person's anus)*				
Fisting (passive)<br>*(Another person's fist enters your anus)*				
Rimming (active)<br>*(Your mouth contacts another person's anus)*				
Rimming (passive)<br>*(Another person's mouth contacts your anus)*				
Use of shared sex toys				
Scat play<br>(*playing with faeces for sexual arousal or activity*)				
Group sex<br>*(Oral and anal sex with two or more partners in a group setting)*				

Q3.3 In the past 3 months, have you used any shared douching equipment before or after sex?

(Douching equipment is used to wash or clean the anus)

- No
- Yes

Part 4: Drug use and sex

This section asks about your experiences of using drugs before or during sex. Please answer the questions as best as you can

Q4.1 In the past 3 months, have you taken any of the following drugs before or during sex? [Tick all that apply]

- Amphetamine (speed)
- Crystal Meth (Tina/Meth/Ice) (smoking/injecting)
- Mephedrone (MCAT/Meow Meow)
- GHB/GBL (Gina/G/Liquid Ecstacy)
- Ketamine (Special K)
- Poppers
- Viagra/Cialis/Levitra (these drugs are known as PDE5i's and are commonly used for erectile enhancement or to treat erectile dysfunction)
- Any other drugs you were not prescribed (please specify)
- None **(Route to Q5)**

Q4.1.1 In the past 3 months, how often have you had sex after taking any of the listed drugs?

- Once
- Two to three times
- More than three times

Q 4.2 In the past 3 months, have you injected any drugs except prescribed medicines or anabolic steroids before or during sex?

- No
- Yes

Part 5: Antibiotic use

Q5.1 In the past 12 months, have you taken antibiotics for any reason, either orally or by injection? (This could be to treat a bacterial STI or for any other infection)

(Please note we will be asking you about antibiotics each week in the follow up questionnaire so if you do receive any treatment in the next three months, please try to record the name of your antibiotic)

- Yes
- No **(Route to 5.3)**

**Q5.2 Which antibiotics have you taken in the past 12 months?** If you do not remember the name of an antibiotic you have taken, please select the option 'Can't remember' and do not try to guess.	**Q5.2.1 When did you take this antibiotic?** Tick multiple boxes if you have taken more than one course of the same antibiotic at different times within the past 12 months (Shown if Q5.2 is ticked)	**Q5.2.2. Which infection did you take this antibiotic for?** Please list all infections, or tick 'can't remember' if you do not remember (Shown if Q5.2 is ticked)
Can't remember<br>Azithromycin<br>Gentamicin<br>Ceftriaxone<br>Ciprofloxacin<br>Doxycycline<br>Penicillin<br>Amoxicillin<br>Tetracycline<br>Moxifloxacin<br>Cefixime<br>Spectinomycin<br>Other [Please specify]	Can't remember<br>Less than 1 week ago<br>1 week to 1 month ago<br>1 to 3 months ago<br>3 to 6 months ago<br>6 to 12 months ago	Can't remember<br>[Enter value]

Q5.3 In the same way that PrEP can be taken to prevent HIV, there is ongoing research to see if antibiotics taken before or shortly after sex protect against STIs such as chlamydia and syphilis (STI prophylaxis). Please note that using antibiotics in this way is not currently recommended by clinicians or public health professionals as sufficient evidence is not yet available and there are concerns that some STIs could become more difficult to treat in the future.

In the past 12 months, have you used antibiotics immediately before or after sex to prevent STIs other than HIV (STI prophylaxis)?

- Yes
- No
- Don't know/can't remember

Part 6: Gastrointestinal Symptoms

Q6.1 Are you currently or have you previously experienced any of the following gastrointestinal or rectal symptoms in the past 3 months? (Tick all that apply)

- Diarrhoea
- Blood in your poo
- Mucus in your poo
- Fever
- Abdominal pain
- Nausea/vomiting
- Loss of appetite
- Other [Please specify]
- None **(Route to Q6.2)**

Q6.1.1 When did these symptoms start?

- Today
- 1-7 days ago
- 1-2 weeks ago
- 2-4 weeks ago
- More than 4 weeks ago

Q6.1.2 How long did these symptoms last?

- Still experiencing symptoms
- Less than 1 week
- 1-2 weeks
- 2-4 weeks
- More than 4 weeks

Q6.2 In the past two weeks have any of your sexual partners had diarrhoea within 7 days (before or after) of sexual contact?

- Yes
- Not that I am aware of
- No sexual partners in last 2 weeks

Q6.3 In the past two weeks have you been in contact with anyone else who had diarrhoea within 7 days (before or after) you had contact with them?

- Yes
- Not that I am aware of

Part 7: Travel

Q7.1 In the past 3 months, have you travelled outside the UK?

- Yes
- No **(Route to end)**

**Q7.2 Where have you travelled?** If you have travelled to multiple locations, or have travelled to the same location on multiple occasions, please enter these in separate boxes	**Q7.2.1 When did you travel to this location?**	**Q7.2.2. Did you have sex with a new partner whilst in this location?**
[Insert value]	Less than 1 week ago<br>1-2 weeks ago<br>2-4 weeks ago<br>More than 4 weeks ago	Yes<br>No

Thank you for completing this questionnaire! Please press the submit button to send your responses
