# Supplementary material for: Acceptability and Feasibility of Longitudinal Sampling for Sexually Transmitted Enteric Infections in Gay, Bisexual, and Other Men Who Have Sex With Men (GBMSM): Prospective Cohort Pilot Study Conducted in 2022 in South East England
Source: JMIR Public Health Surveill. 2026 Mar 30;12:e73762. doi: 10.2196/73762 (PMC13035075; doi:10.2196/73762)
Supplement: Multimedia Appendix 2 [file publichealth-v12-e73762-s002.pdf]

STEIM PILOT STUDY FOLLOW-UP QUESTIONNAIRE (Multimedia appendix)

This is a Multimedia Appendix to a full manuscript published in the J Med Internet Res. For full copyright and citation information see http://dx.doi.org/10.2196/jmir.73762

Thank you for continuing to participate in this study. Please complete this questionnaire once per week on the same day as collecting a rectal swab.

The questionnaire will ask you some personal questions. We are asking these questions because they will help us to better understand how the bugs are spreading between people. You can skip any questions you do not want to answer. All answers are strictly confidential.

Please enter your Study ID:

Please enter today's date:

Part 1: Sexual behaviour

This section asks about your sexual partners and recent sexual history. Please answer the questions as best as you can

Q1.1 Since you provided your last sample, have you had physical sexual contact with any cis men (men assigned male at birth), trans men, trans women or gender-diverse people?

(By sexual contact, we mean any activity intended to achieve orgasm (or close to orgasm) for one or both partners).

- Yes
- No **(Route to Q2)**

Q1.2 Since you provided your last sample, how many men (cis/transgender), trans women or gender-diverse people have you had any physical sexual contact with?

- [Enter value]

Q1.2.1 How many of those partners in Q1.2 were new partners that you had sex with for the first time since you provided your last sample?

- [Enter value]

Q1.2.2 How many of those new partners in Q1.2.1 have you only had sex with once and probably will not have sex with again? (e.g. one-night stands, cruising partner)?

- [Enter value]

We'd now like to ask a bit more detail about the type of sex you have had. Please look at the questions below and answer as best as you can.

	**Q1.3 Since you provided your last sample, what types of sex have you had?** (tick all that apply)	**Q1.3.1 Since you provided your last sample, how often have you used condoms at all times when having this type of sex?** (Shown if Q1.3 is ticked)	**Q1.3.2 Since you provided your last sample, how often have you engaged in this type of sex?** (Shown if Q1.3 is ticked)
Anal sex (insertive/active/top)<br>*(Your penis enters another person's anus)*		Always<br>Sometimes<br>Never	Once<br>Two to three times<br>More than three times
Anal sex (receptive/passive/bottom)<br>*(Another person's penis enters your anus)*			
Receptive anal sex followed by active oral sex<br>*(Another person's penis enters your anus, then your mouth contacts the other persons penis)*			
Insertive anal sex followed by receptive oral sex<br>*(Your penis enters another person's anus, then their mouth contacts your penis)*			
Fisting (active)<br>*(Your fist enters another person's anus)*			
Fisting (passive)<br>*(Another person's fist enters your anus)*			
Rimming (active)<br>*(Your mouth contacts another person's anus)*			
Rimming (passive)<br>*(Another person's mouth contacts your anus)*			
Use of shared sex toys			
Scat play<br>(*playing with faeces for sexual arousal or activity*)			
Group sex<br>*(Oral and anal sex with two or more partners in a group setting)*			

Q1.4 Since you provided your last sample, have you used any shared douching equipment before or after sex?

(Douching equipment is used to wash or clean the anus)

- No
- Yes

Part 2. Drug use and sex

This section asks about your experiences of using drugs before or during sex. Please answer the questions as best as you can

Q2.1 Since you provided your last sample, have you taken any of the following drugs before or during sex? [Tick all that apply]

- Amphetamine (Speed)
- Crystal Meth (Tina/Meth/Ice) (smoking/injecting)
- Mephedrone (MCAT/Meow Meow)
- GHB/GBL (Gina/G/Liquid Ecstacy)
- Ketamine (Special K)
- Poppers
- Viagra/Cialis/Levitra (these drugs are known as PDE5i's and are commonly used for erectile enhancement or to treat erectile dysfunction)
- Any other drugs that you were not prescribed (please specify)
- None **(Route to Q3)**

Q2.1.1 Since you provided your last sample, how often have you had sex after taking any of the listed drugs?

- Once
- Two to three times
- More than 3 times

Q2.2 Since you provided your last sample, have you injected any drugs except prescribed medicines or anabolic steroids before or during sex?

- No
- Yes

Part 3: Antibiotic use

Q3.1 Since you provided your last sample, have you taken antibiotics for any reason, either orally or by injection? (this could be to treat a bacterial STI or for any other infection)

- Yes
- No **(Route to Q3.3)**

**Q3.2. Which antibiotics have you taken since you provided your last sample?** If you do not remember the name of an antibiotic you have taken, please select the option 'Can't remember' and do not try to guess.	**Q3.2.1 Which infection did you take this antibiotic for?** Please list all infections, or tick 'can't remember' if you do not remember<br>(Shown if Q3.2 is ticked)
Can't remember<br>Azithromycin<br>Gentamicin<br>Ceftriaxone<br>Ciprofloxacin<br>Doxycycline<br>Penicillin<br>Amoxicillin<br>Tetracycline<br>Moxifloxacin<br>Cefixime<br>Spectinomycin<br>Other [Please specify)	Can't remember<br>[Enter value]

Q3.3 In the same way that PrEP can be taken to prevent HIV, there is ongoing research to see if antibiotics taken before or after sex protect against STIs such as chlamydia and syphilis (STI prophylaxis). Please note that using antibiotics in this way is not currently recommended by clinicians or public health professionals as sufficient evidence is not yet available and there are concerns that some STIs could become more difficult to treat in the future.

Since providing your last sample, have you used antibiotics immediately before or after sex to prevent STIs other than HIV (STI prophylaxis)?

- Yes
- No
- Don't know/can't remember

Part 4: Gastrointestinal Symptoms

Q4.1 Since providing your last sample, have you experienced any of the following gastrointestinal or rectal symptoms? [Tick all that apply]

- Diarrhoea
- Blood in your poo
- Mucus in your poo
- Fever
- Abdominal pain
- Nausea/vomiting
- Loss of appetite
- Other [Please specify]
- None **(Route to Q4.2)**

Q4.1.1 When did these symptoms start?

- Today
- Less than 1 week ago **(Route to Q4.1.2)**
- More than 1 week ago **(Route to Q4.1.2)**

Q4.1.2 Are you still experiencing symptoms today?

- Yes
- No

Q4.2 Since you provided your last sample, have any of your sexual partners had diarrhoea, either in the week before or after you had sex?

- Yes
- Not that I am aware of
- No sexual partners since last sample

Q4.3 Since providing your last sample, have you been in contact with anyone else who had diarrhoea within 7 days (before or after) you had contact with them?

- Yes
- Not that I am aware of

Part 5: Travel

Q5.1 Since you provided your last sample, have you travelled outside the UK?

- Yes
- No **(Route to end)**

**Q5.2 Where have you travelled?**<br>If you have travelled to multiple locations, or have travelled to the same location on multiple occasions, please enter these in separate boxes	**Q5.2.1 Did you have sex with a new partner whilst in this location?**
[Insert value]	Yes<br>No

Thank you for completing this questionnaire! Please press the submit button to send your responses
