# Supplementary material for: Acceptability and Feasibility of Longitudinal Sampling for Sexually Transmitted Enteric Infections in Gay, Bisexual, and Other Men Who Have Sex With Men (GBMSM): Prospective Cohort Pilot Study Conducted in 2022 in South East England
Source: JMIR Public Health Surveill. 2026 Mar 30;12:e73762. doi: 10.2196/73762 (PMC13035075; doi:10.2196/73762)
Supplement: Multimedia Appendix 3 [file publichealth-v12-e73762-s003.pdf]

STEIM PILOT STUDY CONSENT FORM (Multimedia appendix)

This is a Multimedia Appendix to a full manuscript published in the J Med Internet Res. For full copyright and citation information see http://dx.doi.org/10.2196/jmir.73762

**Title of Project:**	Acceptability and feasibility of longitudinal sample collection to better understand the epidemiology of enteric infections in men who have sex with men (MSM): a pilot study
**IRAS ID:**	304423
**Chief Investigator:**	Dr Hamish Mohammed
**Study ID:**	

Consent for study (required for participation):

Please initial each box if you agree with the statement: You must initial all the boxes in this section to be eligible to take part in the study.

1 I confirm that I have read and understand the information sheet dated 05/01/2022 (version 2.0) for the above study. I have had the opportunity to consider the information, ask questions and have had these answered satisfactorily. ☐

2 I understand that data collected during the study, may be looked at by individuals from the UK Health Security Agency, from regulatory authorities or from the NHS Trust, where it is relevant to my taking part in this research. I give permission for these individuals to have access to my records. ☐

3 I understand that my participation is voluntary and that I am free to withdraw at any time without giving any reason, without my medical care or legal rights being affected. I understand that any samples and data already collected will be retained and used in the study. ☐

4 I agree that any samples I provide may be used for tests for gut bugs and resistance to antibiotics. The samples will be stored securely at the UK Health Security Agency. I understand that I will not be given any results from the tests performed on my samples. ☐

5 I understand that information I provide for this study will be treated as strictly confidential and handled in accordance with the Data Protection Act 2018 and the General Data Protection Regulations (GDPR) ☐

6 I agree to take part in the above study ☐

Consent for additional data collection and linkage (optional):

Please initial each box if you agree with the statement: You do not have to agree to the statements in this section to take part in the study.

1 I consent to the questionnaire responses and test results being linked to existing routinely collected data on STI and HIV tests and diagnoses. ☐

2 I agree to give my contact details (email address or mobile number) to be contacted about sample reminders. ☐

3 I agree to give my contact details (email address or mobile number) to be contacted about taking part in a 1 to 1 interview. ☐

4 I understand that the data and samples I have provided for the study may be stored in an anonymised form and used for future health-related research purposes with appropriate approval from a Research Ethics Committee. ☐

________________	________________	________________
Name of participant	Date	Signature
________________	________________	________________
Name of person taking consent	Date	Signature

When completed: 1 (original) for research site file, 1 (copy) for patient; 1 (copy) to be kept in medical notes.
