# Supplementary material for: Acceptability and Feasibility of Longitudinal Sampling for Sexually Transmitted Enteric Infections in Gay, Bisexual, and Other Men Who Have Sex With Men (GBMSM): Prospective Cohort Pilot Study Conducted in 2022 in South East England
Source: JMIR Public Health Surveill. 2026 Mar 30;12:e73762. doi: 10.2196/73762 (PMC13035075; doi:10.2196/73762)
Supplement: Multimedia Appendix 4 [file publichealth-v12-e73762-s004.pdf]

STEIM pilot study supplementary figures and table

This is a Multimedia Appendix to a full manuscript published in the J Med Internet Res. For full copyright and citation information see http://dx.doi.org/10.2196/jmir.73762

Figure S1. Flowchart of STEIM study recruitment process.

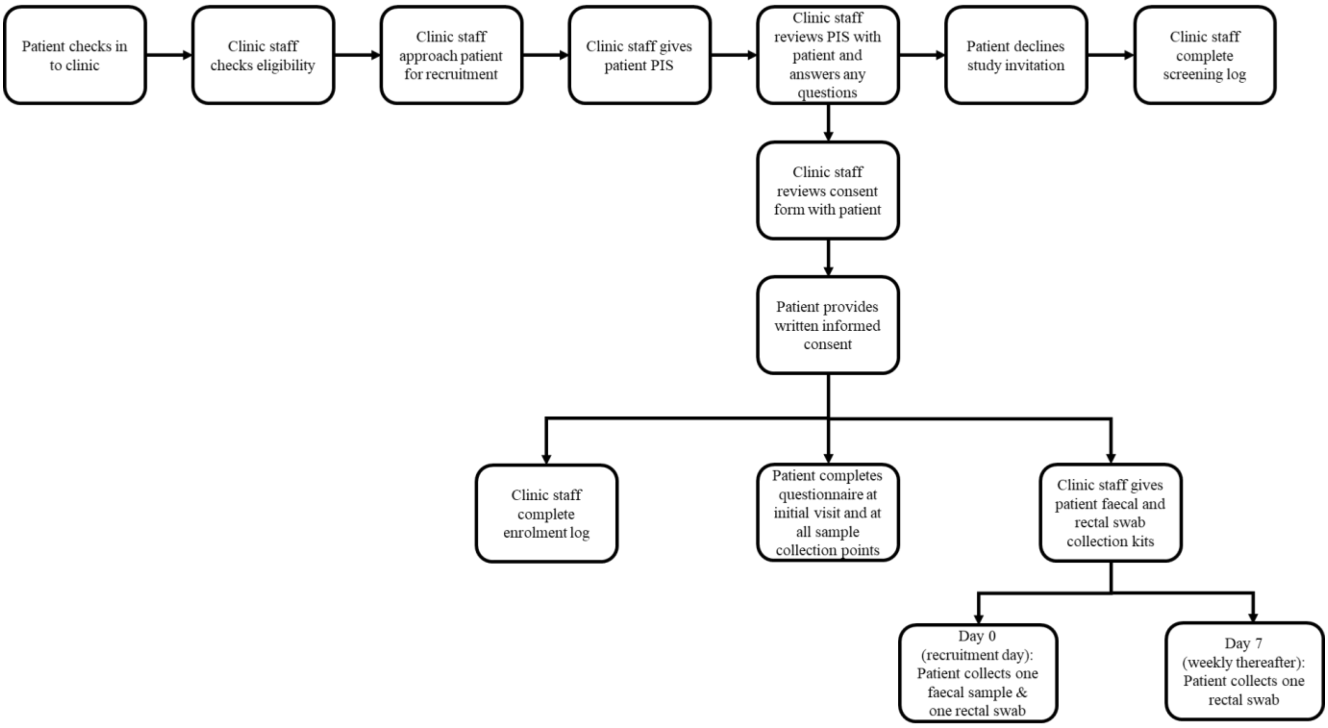

Figure S2. Number of participants returning each swab among those returning at least one swab.

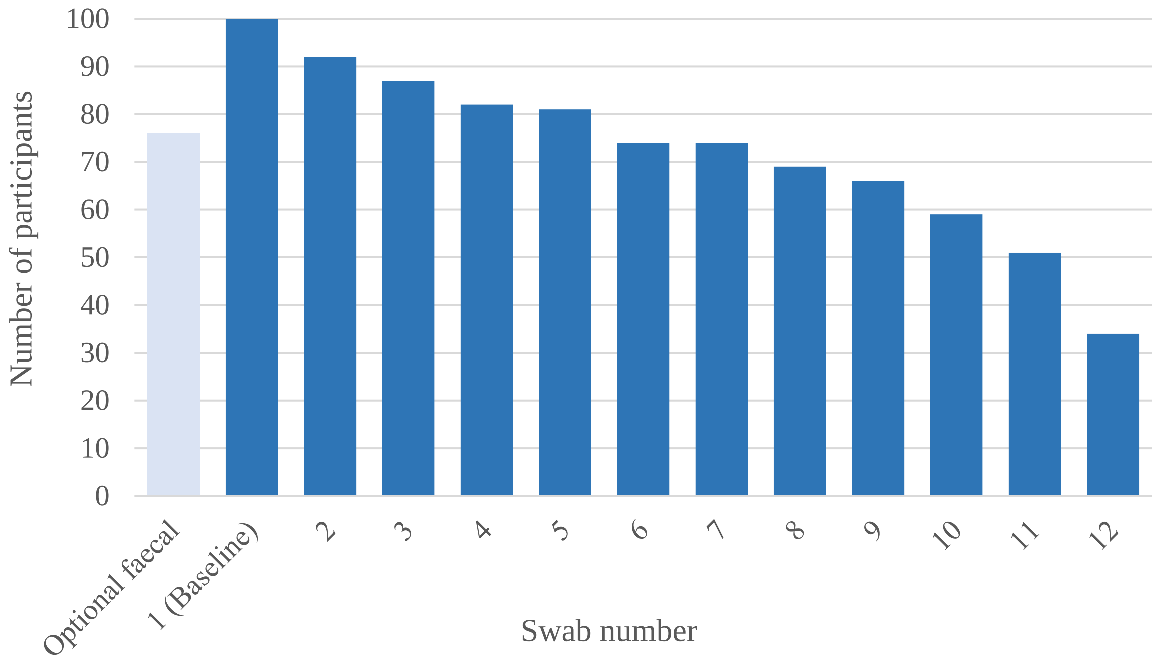

Table S1. Demographic and clinical characteristics among participants who participated and did not participate in the study following recruitment.

	Study participant characteristics who were enrolled and participated (N=99)		Study participant characteristics who were enrolled and did not participate (N=85)		Chi-square test
	n	%	n	%	*P* value
**Age, in years**					
16 to 24	2	2.0	4	4.7	0.32
25 to 34	21	21.2	26	30.6	
35 to 44	24	24.2	20	23.5	
45 to 64	46	46.5	33	38.8	
65 and over	6	6.1	2	2.4	
Unknown/missing	0	0	0	0	
**Sexual orientation**					
Heterosexual	1	1.0	0	0.0	0.34
Gay	91	91.9	73	85.9	
Bisexual	3	3.0	6	7.1	
Other, not listed	4	4.0	6	7.1	
Unknown	0	0.0	0	0.0	
**Ethnicity[a]**					
White	92	92.9	79	92.9	0.59
All other ethnic groups	4	4.0	5	5.9	
Unknown/missing	3	3.0	1	1.2	
**Region of Birth[b]**					
UK	75	75.8	66	77.6	0.76
All other regions of birth	24	24.2	19	22.4	
Unknown	0	0.0	0	0.0	
**Index of multiple deprivation (IMD)**					
Most deprived	14	14.1	20	23.5	0.004
2	22	22.2	21	24.7	
3	25	25.3	27	31.8	
4	22	22.2	12	14.1	
Least deprived	15	15.2	1	1.2	
Unknown/missing	1	1.0	4	4.7	
**HIV status with PrEP status**					
HIV negative or unknown status, taking PrEP	46	46.5	18	21.2	0.001
HIV negative or unknown status, not taking PrEP	27	27.3	43	50.6	
Living with HIV	26	26.3	24	28.2	
**STI in previous year**					
Yes	39	39.4	31	36.5	0.68
No	60	60.6	54	63.5	
**Concurrent STI (i.e. STI on day of enrolment)[c]**	**(N=95)**		**(N=84)**		
Yes	2	2.1	7	8.3	0.057
No	93	97.9	77	91.7	

Table footnotes

[a]All other ethnic groups includes groupings for those of Asian ethnicity, black ethnicity, mixed ethnicity, and any other ethnicity. This data was aggregated into one group due to the presence of small data points.

[b]All other regions of birth includes groupings for those born in the Caribbean, Central and South America, North America, Europe, South Asia, Sub-Saharan Africa, and any other region. This data was aggregated into one group due to the presence of small data points.

[c]Concurrent or previous STI was considered as *Chlamydia trachomatis*, *Neisseria gonorrhoeae* or *Treponema pallidum* (primary, secondary, or early latent) diagnosis.
